# Vonoprazan-based triple therapy is non-inferior to susceptibility-guided proton pump inhibitor-based triple therapy for *Helicobacter pylori* eradication

**DOI:** 10.1186/s12941-018-0281-x

**Published:** 2018-06-28

**Authors:** Hiroki Tanabe, Keiichi Yoshino, Katsuyoshi Ando, Yoshiki Nomura, Katsuhisa Ohta, Kiichi Satoh, Eiichiro Ichiishi, Akiei Ishizuka, Takaaki Otake, Yutaka Kohgo, Mikihiro Fujiya, Toshikatsu Okumura

**Affiliations:** 1 Department of Gastroenterology, International University of Health and Welfare Hospital, Iguchi 537-3, Nasushiobara, Tochigi 329-2763 Japan; 2 Division of Gastroenterology and Hematology/Oncology, Department of Medicine, Asahikawa Medical University, Midorigaoka-Higashi 2-1-1-1, Asahikawa, Hokkaido 078-8510 Japan; 3 Present Address: Jiseikai-Kamiitabashi Hospital, 4-36-9 Tokiwadai Itabashi-ku, Tokyo, 174-0071 Japan

## Abstract

**Background:**

All *Helicobacter pylori*-infected patients are recommended for eradication with an appropriate regimen in each geographic area. The choice of the therapy is somewhat dependent on the antimicrobial susceptibility. The rate of clarithromycin resistance has been increasing and is associated with failure; thus, susceptibility testing is recommended before triple therapy with clarithromycin. However, antimicrobial susceptibility testing is not yet clinically available and an alternative newly developed acid inhibitor vonoprazan is used for triple therapy in Japan. The aim of this study was to determine whether vonoprazan-based triple therapy is plausible treatment in *H. pylori* eradication.

**Methods:**

A retrospective observational study of *H. pylori* eradication was conducted in a single institute. The patients who requested antimicrobial susceptibility testing were treated with susceptibility-guided proton pump inhibitor-based triple therapy in International University of Health and Welfare Hospital from 2013 to 2016. Other patients were treated with empirical treatment with a proton pump inhibitor. From 2015 to 2016, vonoprazan-based triple treatment (vonoprazan, 20 mg; amoxicillin, 750 mg; and clarithromycin, 200 or 400 mg, b.i.d.) was conducted, and its effectiveness was compared with susceptibility-guided proton pump inhibitor-based triple therapy. We also investigated the improvement in eradication rate when antimicrobial susceptibility testing was performed, and compared the outcomes of vonoprazan-based and proton pump inhibitor-based empirical therapy.

**Results:**

A total of 1355 patients who received first-line eradication treatment were enrolled in the present study. The eradication rates of the empirical proton pump inhibitor-based therapy and the vonoprazan-based therapy group in a per-protocol analysis were 86.3% (95% CI 83.8–88.8) and 97.4% (95% CI 95.7–99.1), respectively. In 212 patients who received antimicrobial susceptibility testing, the rate of clarithromycin resistant was 23.5% and the eradication rate in susceptibility-guided treatment was 95.7% (95% CI 92.9–98.4). The difference between susceptibility-guided and vonoprazan-based therapy was − 1.7% (95% CI − 4.9 to 1.5%), and the non-inferiority of vonoprazan-based triple therapy was confirmed.

**Conclusions:**

Vonoprazan-based triple therapy was effective as susceptibility-guided triple therapy for *H. pylori* eradication. An empirical triple therapy with vonoprazan is preferable even in area with high rates of clarithromycin-resistance.

*Trial registration* The study was retrospectively registered in University Hospital Medical Information Network (UMIN000032351)

## Background

*Helicobacter pylori* (*H. pylori*) infection is a cause of non-cardiac gastric cancer and its eradication may reduce the risk of by 40% [1]. It is recommended that *H. pylori*-infected patients receive eradication therapy, and for decades, various *H. pylori* eradication regimens have been investigated around the world. No single standard therapy is recommended because situations such as host- or bacteria-related factors are different in each area. The first Maastricht conference proposed initial triple therapy including a proton pump inhibitor (PPI), clarithromycin and amoxicillin or metronidazole [2]. However, recent data shows that the eradication rate with the triple therapy is < 80% [3]. There are several explanations for the decrease: low medicine compliance, high intra-gastric acidity, a high bacterial load, and the strain variability. The most important cause is the emergence of antibiotic resistance in *H. pylori*. Moreover the rate of clarithromycin resistance is dramatically increasing in North America, Europe, and East Asia [4, 5]. Maastricht IV recommends that the triple therapy should be abandoned when the rate of clarithromycin resistance in the region is > 15–20% [6]. Antimicrobial susceptibility testing is weakly recommended before standard triple therapy in the recent Maastricht V consensus report [7]. A meta-analysis of randomized controlled trials (RCTs) shows that culture-guided triple therapy is superior based on the higher eradication rate and lower cost [8].

Population-based antimicrobial susceptibility testing may be transferred to individual-based susceptibility testing [7]. In areas of low clarithromycin resistance, triple therapy with PPI, clarithromycin, and amoxicillin is recommended as a first-line empirical treatment. In areas of high clarithromycin resistance, the choice of therapy is based on metronidazole resistance. In the areas of very low metronidazole resistance, it is recommended that metronidazole be replaced by clarithromycin in triple therapy. In regions with low to intermediate metronidazole resistance, non-bismuth quadruple therapy (sequential therapy or concomitant therapy) is proposed as a first-line treatment. An RCT in Taiwan revealed that bismuth quadruple therapy was superior to triple therapy [9].

The newly discovered acid inhibitory reagent, vonoprazan is used in *H. pylori* eradication treatment [10]. Vonoprazan, a newly developed potassium-competitive acid blocker (P-CAB), strongly inhibit the secretion of gastric acid from the parietal cells in the stomach, and vonoprazan-based triple therapy was expected to be more effective than PPI-based triple therapy. An RCT comparing vonoprazan and lansoprazole showed that vonoprazan-based triple therapy with amoxicillin and clarithromycin is effective with a high eradication rate of > 90% [11]. The therapy was shown to be effective even for clarithromycin-resistant individuals (eradication rate; 82.0%). Based on these findings, vonoprazan-based triple therapy with amoxicillin and clarithromycin was approved in Japan.

The old regimen, the bismuth-containing quadruple therapy and the new vonoprazan-based regimens are currently proposed for the eradication of *H. pylori*. In our country, vonoprazan is available but bismuth is not. We therefore conducted a comparative study of susceptibility-guided PPI-based triple therapy versus vonoprazan-based triple therapy for *H. pylori* eradication.

## Methods

### The patients and study protocol

This was a retrospective observational study of *H. pylori*-infected patients who received first-line eradication treatment in University of Health and Welfare Hospital from February 2013 to February 2016. The patients were classified into three groups: the standard PPI-based triple therapy group, the antimicrobial susceptibility-guided triple therapy group, and the vonoprazan-based triple therapy group. PPI-based triple therapy was performed for the patients who were treated from 2013 to 2015, while vonoprazan-based triple therapy was performed from 2015 to 2016. This study was conducted in accordance with the principles of the Declaration of Helsinki and the study protocol was approved by the Institutional Review Board of International University of Health and Welfare Hospital (#13-B-152). The study was retrospectively registered in the University Hospital Medical Information Network (UMIN000032351).

### Eradication treatment

The presence of *H. pylori* was diagnosed in clinical practice by at least one of the following methods: a rapid urease test, a ^13^C-urease breath test, an *H. pylori* immunoglobulin G serological test, or an *H. pylori* stool antigen test. Triple therapy using PPI, amoxicillin, and clarithromycin is recommended by the Japanese guidelines [10]. The PPI-based regimen was PPI (lansoprazole, 30 mg; rabeprazole, 10 mg; or esomeprazole, 20 mg, b.i.d.) with amoxicillin (750 mg, b.i.d.), and clarithromycin (200–400 mg, b.i.d.) for 7 days. In the regimen for vonoprazan-based therapy, vonoprazan (20 mg b.i.d.) was substituted for PPI-based therapy. The success of eradication was determined by a ^13^C-urea breath test (< 0.25%) or an *H. pylori* stool antigen test (negative).

### Antimicrobial susceptibility testing

Susceptibility testing was performed according to the physician’s preference and at the patient’s request. A written informed consent was obtained from each patient before performing endoscopy. For each patient, two biopsies were taken from the gastric antrum and corpus for bacterial culturing. After *H. pylori* was cultured, susceptibility testing was performed by the agar dilution method. In susceptibility testing, the minimum inhibitory concentrations (MICs) of amoxicillin, clarithromycin, and metronidazole were measured. Microbial resistance was defined as MIC > 1 µg/ml to clarithromycin and amoxicillin and > 16 µg/ml to metronidazole. Clarithromycin-susceptible *H. pylori* was treated with a PPI and amoxicillin (750 mg, b.i.d.), clarithromycin (200–400 mg b.i.d.) for 7 days (PPI-AC). Resistant *H. pylori* was treated with a PPI with amoxicillin (750 mg, b.i.d.) and metronidazole (250 mg, b.i.d.) for 7 days (PPI-AM).

### The outcomes and statistical analysis

The primary endpoint was the non-inferiority of vonoprazan-based treatment to susceptibility-guided therapy. The eradication rate was determined by an intention-to treat (ITT) and a per-protocol (PP) analysis, and a PP analysis was used for the comparisons between the groups. Eradication rates were estimated to 90% in previous reports [11]. The sample size was estimated to ensure a statistical power of 90% in the detection of non-inferiority margin of 10% at a significance level of 2.5%. The ratio of the susceptibility-guided PPI-based group to the vonoprazan-based group was 1:2. A total of 427 patients (143 patients in the susceptibility-guided PPI-based group, and 284 in the vonoprazan-based group) were required to detect the non-inferiority of vonoprazan-based eradication therapy.

The secondary endpoint was the superiority of susceptibility-guided therapy or vonoprazan-based therapy to empirical PPI-based treatment. The eradication rates were estimated to 90% in the clarithromycin-susceptibility-guided therapy and the vonoprazan-based therapy group, and 85% in the empirical PPI-based therapy group. The sample size was estimated to ensure a statistical power of 80% with an alpha value of 5% in the detection of superiority. A total of 1080 patients (540 in each group) were required to detect significant difference with a Chi square test.

## Results

**Fig. 1:**
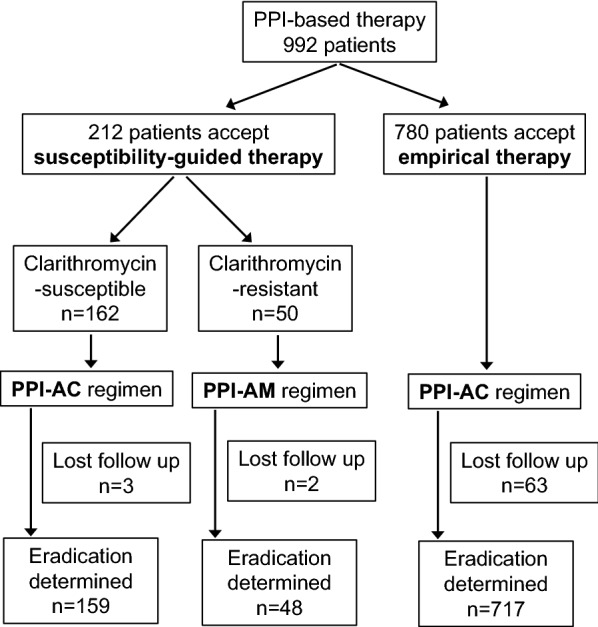
A flow diagram of proton pump inhibitor-based therapy. Patients who underwent clarithromycin susceptibility testing were treated by either PPI-AC or PPI-AM regimen. PPI-AC therapy was chosen for empirical therapy. *PPI-AC* proton pump inhibitor with amoxicillin and clarithromycin, *PPI-AM* proton pump inhibitor with amoxicillin and metronidazole

**Fig. 2:**
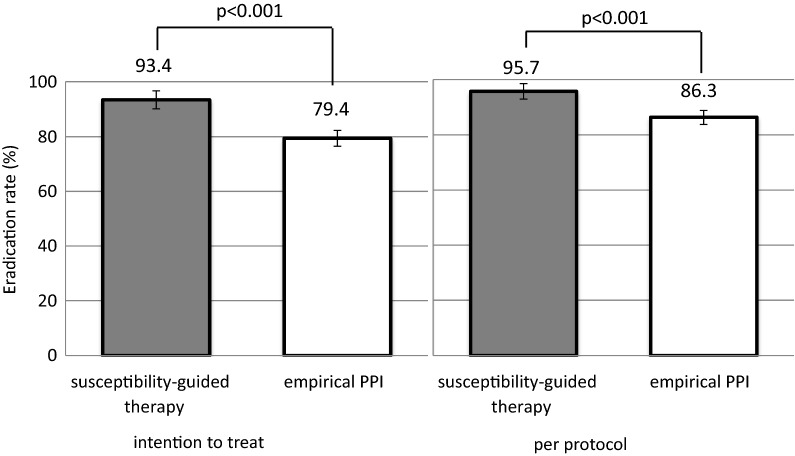
The *Helicobacter pylori* eradication rates in susceptibility-guided therapy and empirical therapy. The *p*-values for superiority in susceptibility-guided proton pump inhibitor-based triple therapy are provided. *PPI* proton pump inhibitor

A total of 1355 patients were enrolled in this study. A total of 992 patients received PPI-based therapy; 780 received PPI-based empirical therapy and 212 received susceptibility-guided therapy (Fig. 1). Among the patients who accepted antimicrobial susceptibility testing, the rate of clarithromycin resistance was 23.5% (Table 1). The rates of metronidazole and amoxicillin resistance were 4.2 and 0%, respectively, and neither was considered when selecting an eradication regimen in this study. One hundred sixty-two clarithromycin-susceptible patients were treated by a PPI-AC regimen and 50 clarithromycin-resistant patients were treated using a PPI-AM regimen. The eradication success rates of susceptibility-based eradication therapy are shown in Table 2. The eradication rates of PPI-AC in clarithromycin-susceptible cases were 93.8% (95% CI 90.1–97.5) in the ITT analysis and 95.6% (95% CI 92.6–98.8) in the PP analysis. The total eradication rates of the susceptibility-guided therapy group were 93.4% (95% CI 90.1–96.7) in the ITT analysis and 95.7% (95% CI 92.9–98.4) in the PP analysis (Fig. 2). Only 2.3% difference was observed between the ITT and PP analysis. Four of the nine metronidazole-resistant patients were susceptible to clarithromycin and were treated by PPI-AC. The other five were resistant to clarithromycin but were treated by PPI-AM. Eradication was successfully achieved in all patients; one patient was not followed until an eradication test.

**Table 1:**
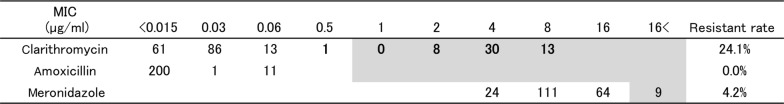
The minimal inhibitory concentration of cultured *Helicobacter pylori*

Data are expressed as number of *Helicobacter pylori*. The number in gray zone indicates above minimal inhibitory concentrations (MIC)

**Table 2:** The eradication rates of susceptibility-guided PPI-based therapy

	Clarithromycin susceptible n = 162	Clarithromycin resistant n = 50	Total n = 212	Success rate (%) (95% confidence interval)
Intention to treat	152/162	46/50	198/212	93.4 (90.1–96.7)
Per protocol	152/159	46/48	198/207	95.7 (92.9–98.4)

For empirical therapy, 780 patients who received PPI-based therapy and 363 who received vonoprazan-based therapy did not request susceptibility testing prior to eradication treatment (Table 3). The success rates of the PPI-based and vonoprazan-based regimens in the PP analysis were 86.3% (95% CI 83.8–88.8) and 97.4% (95% CI 95.7–99.1), respectively. The vonoprazan-based therapy was significantly more effective (*p *< 0.001) than PPI-based therapy. Eradication treatment was discontinued due to adverse events by five patients receiving PPI-based therapy (vomiting, n = 2; dermatitis, n = 2; diarrhea, n = 1) and three patients receiving vonoprazan-based therapy (dermatitis, n = 1; vomiting, n = 1; taste disturbance, n = 1).

**Table 3:** The eradication rates of empirical eradication therapy

	PPI + AC				P − CAB + AC vonoprazan n = 363
	Esomeprazole n = 123	Rabeprazole n = 450	Lansoprazole n = 207	Total n = 780	
Intention to treat	100/123	359/450	160/207	619/780	332/363
% (95% CI)	81.3 (74.4–88.2)	79.8 (76.1–83.5)	77.3 (71.6–83.0)	79.4 (76.5–82.2)	91.5 (88.6–94.3)
Per protocol	100/113	359/417	160/187	619/717	332/341
% (95% CI)	88.5 (82.6–94.4)	86.1 (82.8–89.4)	85.6 (80.5–90.6)	86.3 (83.8–88.8)	97.4 (95.7–99.1)

*PPI *+ *AC* proton pump inhibitor with amoxicillin and clarithromycin, *P* − *CAB *+ *AC* potassium-competitive acid blocker with amoxicillin and clarithromycin

**Fig. 3:**
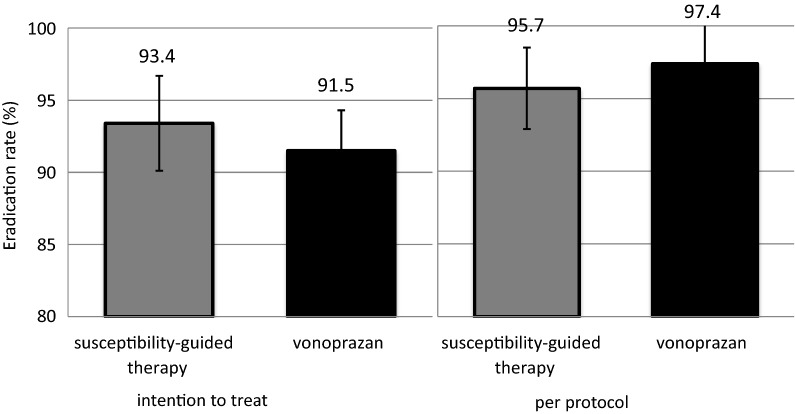
The *Helicobacter pylori* eradication rates in susceptibility-guided therapy and empirical vonoprazan-based triple therapy. The differences in the eradication rates between these two therapies (susceptibility-guided therapy minus vonoprazan-based therapy) were 1.9% in the intention-to-treat analysis and − 1.7% in the per-protocol analysis

The primary endpoint, the inferiority of the vonoprazan-based therapy to susceptibility-guided PPI-based therapy, was verified. The success rates of the susceptibility-guided PPI-based therapy and the vonoprazan-based therapy in the PP analysis were 95.7% (95% CI 92.9–98.4) and 97.4% (95% CI 95.7–99.1), respectively (Fig. 3). The difference between these therapies (susceptibility-based minus vonoprazan-based) was − 1.7% (95% CI − 4.9 to 1.5%). Vonoprazan-based therapy was shown to be non-inferiority to susceptibility-guided PPI-based therapy with a non-inferiority margin of 10%.

## Discussion

This is the first comparative study of the efficacy of susceptibility-guided PPI-based triple therapy and vonoprazan-based triple therapy in the *H. pylori* eradication therapy. Antibiotic resistance is one of the major causes of the eradication failure [12]. It is recommended that the regional antibiotic resistance patterns to be determined and that an adequate regimen be selected for successful eradication. Several susceptibility tests, such as culture-based or mutation analyses are available [13–16]. Some RCTs have compared the efficacy of eradication treatment with or without culture-based guidance and a meta-analysis have concluded that culture-guided triple therapy was more effective in the economic costs and benefits than standard triple therapy for first-line treatment [8, 17, 18]. Our study confirmed that the eradication rate of susceptibility-guided PPI therapy was significantly higher than that of empirical PPI-AC therapy. The disadvantage of culture-based susceptibility testing is that it is a time consuming task that delay the start of eradication therapy. Many patients refused antibiotics sensitivity testing before eradication therapy in our series (Fig. 1). Some possible reasons for this include the fact that antibiotic sensitivity testing is not yet covered by national health insurance, endoscopic examination is required for *H. pylori* culturing and the subsequent MIC measurement, and patients need an additional medical consultation for the diagnosis. Thus, susceptibility testing is not available for all patients in the clinical setting. A novel eradication regimen that is effective irrespective of the antimicrobial resistance is desired. A novel finding in our study is that an empirical eradication strategy with a vonoprazan-based triple regimen was not inferior to tailored therapy with a PPI-based triple regimen.

In North America, where there is a high clarithromycin resistance, bismuth quadruple therapy consisting of a PPI, bismuth, tetracycline, and metronidazole for 10–14 days has shown a high success rate and is recommended as a first-line treatment [4]. However, bismuth is not available in Japan. Either non-bismuth concomitant therapy or sequential therapy is recommended as an alternative. For Japanese patients, vonoprazan-based triple therapy has recently become available. The regimen for the first-line therapy is vonoprazan (20 mg), amoxicillin (750 mg), clarithromycin (200–400 mg, b.i.d.) for 7 days, and metronidazole (250 mg) instead of clarithromycin for second-line therapy. Some reports from Japan have presented eradication rates as high as 90% [19–22]. In our study, the success rate on vonoprazan-based triple therapy was 91.5% in the ITT analysis and 97.4% in the PP analysis, which is extremely high. Judging from the antimicrobial resistance in our hospital (Table 1), clarithromycin sensitivity (resistance rate, 24.1%) may be the reason for the high efficacy. Matsumoto et al. [23] and Murakami et al. [11] reported much higher resistance rates (44.7 and 30.4%) and lower eradication rates (89.6% in the PP analysis and 92.6% in full analysis set), respectively. In the area of the high clarithromycin-resistance (> 15%), vonoprazan with amoxicillin and metronidazole may be an option for empirical therapy. A new regimen with vonoprazan would still need to be tested in different regions around the world.

In our retrospective study, the sample size of the patients was estimated in advance for the statistical analysis. The primary endpoint with regard to demonstrating the non-inferiority of vonoprazan-based therapy in comparison to susceptibility-guided PPI therapy required 284 and 143 patients, respectively. Eventually, 1355 patients were enrolled, 363 of whom were treated with vonoprazan-based therapy and 212 who were treated with susceptibility-guided therapy and sufficient numbers of participants were analyzed. The 780 patients who underwent PPI-based treatment were also included to investigate the secondary endpoint. The number of participants included in the comparison of susceptibility-based therapy and PPI-based therapy was 992, which was less than 1080 that was required (according to our estimation). The statistical analysis of this study population revealed a significant difference between the two groups (p < 0.001) (Fig. 2).

The present study was associated with some limitations. First, our study was an observation study that was performed in a single institute. The total number of patients was greater than that was required for the analysis of the primary endpoint, according to our estimate; however there were fewer participants in the investigation of antimicrobial susceptibility testing. Greater numbers of the patients were included in the standard PPI-based triple therapy group than the culture-guided group. Second, in the present study, the antibacterial susceptibility patterns from the patients in the vonoprazan-based treatment group were not tested. In phase III trial of vonoprazan-based triple therapy [11], the eradication rate was 82.0%, even for clarithromycin-resistant *H. pylori*, however lower eradication rates were estimated [23]. It is crucial to determine the efficacy of a vonoprazan-based regimen with or without culture-based therapy. The selection of sensitive antimicrobials (e.g. metronidazole and amoxicillin) should be considered for clarithromycin-resistant *H. pylori*. Third, the PPI-based regimen conducted in our study was administered for 7 days, which is covered by national health insurance. A longer therapy is recommended to obtain a sufficient eradication rate in patients with triple therapy [24]. A 10–14 days eradication regimen that include vonoprazan with antimicrobials should be tested in Western countries. Consequently, there are still some possibilities to improve the eradication success rate in patients treated with vonoprazan-based regimens.

## Conclusions

Clarithromycin-susceptibility testing of *H. pylori* showed that the resistance rate in the geographical area was 24.1% and susceptibility-guided triple therapy achieved a very high eradication rate. The eradication rate achieved with vonoprazan-based triple therapy was as the eradication rate achieved with susceptibility-guided triple therapy. Prior susceptibility testing can be avoided and prompt eradication with vonoprazan-based therapy is preferable in area even with a high rate of clarithromycin resistance.

## Abbreviations

*H. pylori*: *Helicobacter pylori*
PPI: proton pump inhibitor
RCT: randomized controlled trial
P-CAB: potassium-competitive acid blocker
MIC: minimum inhibitory concentration
PPI-AC: proton pump inhibitor with amoxicillin and clarithromycin
PPI-AM: proton pump inhibitor with amoxicillin and metronidazole
ITT: intention-to-treat
PP: per-protocol
CI: confidence interval
